# Distributed Broadcast Control of Multi-Agent Systems Using Hierarchical Coordination

**DOI:** 10.3390/biomimetics9070407

**Published:** 2024-07-05

**Authors:** Mahmudul Hasan, Mohammad Khalid Saifullah, Md Abdus Samad Kamal, Kou Yamada

**Affiliations:** Division of Mechanical Science and Technology, Graduate School of Science and Technology, Gunma University, Kiryu 376-8515, Japan; t242b006@gunma-u.ac.jp (M.H.); t221b607@gunma-u.ac.jp (M.K.S.); yamada@gunma-u.ac.jp (K.Y.)

**Keywords:** broadcast control, stochastic optimal control problems, decentralized control, coverage of multi-agent

## Abstract

Broadcast control (BC) is a bio-inspired coordination technique for a swarm of agents in which a single coordinator broadcasts an identical scalar signal to all performing agents without discrimination, and the agents make appropriate moves towards the agents’ collective optimal state without communicating with one another. The BC technique aims to accomplish a globally assigned task for which BC utilizes a stochastic optimization algorithm to coordinate a group of agents. However, the challenge intensifies as the system becomes larger: it requires a larger number of agents, which protracts the converging time for a single coordinator-based BC model. This paper proposes a revamped version of BC model, which assimilates distributed multiple coordinators to control a larger multi-agent system efficiently in a pragmatic manner. Precisely, in this hierarchical BC scheme, the distributed multiple sub-coordinators broadcast the identical feedback signal to the agents, which they receive from the global coordinator to accomplish the coverage control task of the ordinary agents. The dual role of sub-coordinators is manipulated by introducing weighted averaging of the gradient estimation under the stochastic optimization mechanism. The potency of the proposed model is analyzed with numerical simulation for a coverage control task, and various performance aspects are compared with the typical BC schemes to demonstrate its practicability and performance improvement. Particularly, the proposed scheme shows the same convergence with about 30% less traveling costs, and the near convergence is reached by only about one-third of iteration steps compared to the typical BC.

## 1. Introduction

Swarm robotics is a strategy for navigating a large number of non-smart agents to accomplish tasks that are arduous for single agents or humans [1,2].Inspired by natural multi-agent systems like ant colonies, bird flocks, and the collective foraging behavior shown by fish schools, scientists strive to develop innovative algorithms and control mechanisms for effective cooperation in robotic systems [3,4,5,6,7,8,9,10]. Even in the real world, multi-agent systems are being used in numerous sophisticated instances, such as controlling unmanned aerial vehicles (UAVs), unmanned ground vehicles (UGVs), unmanned underwater vehicles (UUVs), etc. to the extent of multi-robot surveillance, planetary exploration, search and rescue missions, service robots in smart homes and offices, warehouse management, and transportation [11]. Examined and employed across a spectrum of disciplines, multi-agent systems have found practical applications in cooperative mobile robotics [12], distributed artificial intelligence [13], social studies [14], biology [15], traffic control [16,17], and supply chain management [18]. The versatility of these systems enables their use in various scenarios, thus contributing to advancements in numerous fields. Drones, in the context of swarm robotics, are rooting multifarious innovative commercialized applications such as impromptu response, surveillance, parcel dispatch, aerial photo–videography, and so on [19].

The realm of collaborative control for multi-agent systems is formulated through diverse methodologies, including optimal control approach [20], the path planning approach [21], and the heuristic approach [22]. The collective behavior of robot swarms is governed by swarm intelligence, which results from local interactions among individual robots and their environment [23]. Existing control methods and algorithms for mobile multi-agent systems or vehicles often rely on communication between all agents [24]. Multi-agent control system requires information about its neighboring agents, thus requiring dimensional and terrestrial computations before devising decisions and executing actions. Common control techniques for multi-agent systems include centralized, decentralized, and distributed approaches [25]. For the purpose of designing the controlling mechanism of multi-agent systems or vehicles, communication among all agents is crucial in most existing control methods.

Some researchers have proposed broadcast control (BC) as an alternative coordination approach for autonomous agents with flexible options to choose any action and achieve collective goals, which utilizes the one-to-all communication layout to navigate the multi-agent system [26]. Originally inspired by Ueda, this framework was initially applied to regulate a bio-inspired actuator system with numerous cellular units [27]. The BC concept has been implemented not only in biological systems but also in the coordination of multi-agent groups, linked automated vehicles on highway merging, unmanned aerial vehicles, and radar surveillance systems [26,28,29,30,31,32,33]. The BC algorithm [26] integrates centralized and decentralized models, thus eliminating the need for explicit communication among agents [34]. In this model, all agents indiscriminately receive identical signals from the supervisor. The BC scheme operates stochastically to optimize given tasks, with the supervisor evaluating the overall performance by observing the agents and uniformly sending commands [35]. Several studies have extended the BC framework to address quantized environments [32], instability issues [36], Markovian environments [31], and even consensus problems [33] with agent-to-agent communication in a broadcast-mixed environment.

The BC framework has found applications in solving motion coordination tasks for multi-agent systems, particularly addressing the coverage problem. Cortes proposed the Voronoi tessellation-based coverage control (VTCC), which involves dividing the coverage area into Voronoi regions assigned to individual robots [37]. The robots move towards the center of gravity of their allocated regions, with the overall swarm cost function decreasing over time, thus ensuring appropriate spatial distribution [38]. This method, which is widely used for its stability guarantees and algorithm simplicity, interprets coverage control as an optimal transport problem [39]. In contrast, the one-to-all communication framework, adopted for practical motion coordination tasks in large-scale multi-robot systems like swarm robots, involves broadcasting a uniform signal to all robots without discrimination [35]. While advantageous, this approach requires agents to be attentive to the spatial and temporal state of neighboring agents, thus resulting in substantial computational data and potential mission jeopardy [34].

The BC framework, introduced by Azuma, utilizes a stochastic optimization method to reduce communication volume without agent-to-agent communication, which has been demonstrated to be effective through numerical simulations and experiments for a small-sized multi-agent system of seven robots in a squared area [26]. Pseudo-perturbation-based BC (PBC), proposed by Ito et al., improves control performance by employing multiple virtual random actions. PBC minimizes unavailing actions, converges states more rapidly than BC, and has applications in tasks such as traffic merging on roads [35]. The work presented in this paper is inspired by the BC framework, particularly its application to multi-agent systems, thus showcasing its potential in solving various coordination tasks with efficiency and stability.

The BC scheme encounters challenges in large-area coverage, thus exhibiting decreased operational efficiency with additional agents, as many iterations and high execution costs are required for extensive coverage. Consequently, the surplus iterations may lead to prolonged convergence time that is expected to leave substantial portions of the large area under lax surveillance [35]. Specifically, the number of decision variables increases with the size of a multi-agent system, and like any optimization with polynomial complexity, the convergence of BC as stochastic programming for a large size is expected to be even harder [40]. In response to these challenges, we propose a multiple-coordinator-based distributed BC scheme for multi-agent tasks. This scheme utilizes multiple agents as coordinators based on the area’s scale, with hierarchical leadership transfers to guide local agents, thus expecting more efficient and enhanced coverage. Specifically, we considered a realistic multi-agent coverage task, where a large group of agents initially positioned in one corner of the area must disperse with a restricted moving distance per step under a scaled coordination task. As the first-ever realistic approach of its kind, we propose a hierarchical coordination task by introducing a global coordinator to control basic coverage comprising a few selected agents as sub-coordinators (SCs). Each SC, with a limited visual or control range, independently provides local (or partial) coverage feedback to the surrounding agents. Ordinary agents receive feedback from the nearby SCs and repeatedly determine their move to achieve global coverage while working locally. In this way, the size of the hierarchical coordination tasks of the GC or SCs is limited in terms of the respective number of agents for realistic implementation. A stochastic gradient estimation method using a weighted average technique for multiple feedback values and a momentum-based smoothing technique to overcome any noisy feedback (due to partial view) were introduced for ordinary agents. The proposed distributed broadcast control with and without the momentum factor was numerically evaluated, and convergences and other performances were compared with the existing broadcast control. The key contributions of this paper are summarized as follows.

- We developed a novel distributed BC scheme in a hierarchical framework to efficiently control a multi-agent system using multiple sub-coordinators.
- We formulated a weighted average gradient estimation technique for ordinary agents to act under local SCs optimally to accomplish the global task.
- We demonstrated that DBC can accomplish a benchmark coverage problem more cost-effectively and faster than typical BC for large multi-agent systems.

The rest of the paper is organized as follows. Section 2 describes the proposed distributed broadcast control scheme with a hierarchical strategy and control decision mechanism after reviewing the basic broadcast control using a single coordinator. Section 3 describes the numerical simulation to compare and evaluate the proposed scheme, and Section 4 concludes the outcomes, thus providing a direction for future enhancement.

## 2. Distributed Broadcast Control Scheme

This paper proposes a distributed BC scheme, henceforth referred to as the DBC scheme for brevity, of multi-agent systems and illustrates its concept, formulation, and evaluation using a standard coverage control problem. At first, in this section, the single-coordinator-based typical control problem, used in [26,35], is reviewed better to understand the concept of the proposed DBC scheme.

### 2.1. Single-Coordinator-Based BC Scheme

**Figure 1:**
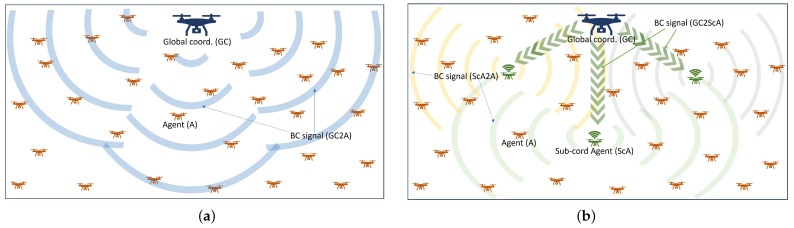
Fundamental concept of (**a**) typical single-coordinator-based BC scheme and (**b**) distributed BC scheme. The BC signal wave is transmitted from (**a**) the global coordinator to the ordinary agents (blue waves) and (**b**) the global coordinator to the sub-coordinator agents (the green arrow line); then, the sub-coordinator agents transmit the BC signals to the ordinary agents (gray, green, and orange waves).

The typical broadcast control scheme is illustrated in Figure 1a, where a global coordinator broadcasts the same signal to all agents in the environment based on their task achievement score or objective value. Let us assume that there are *N* agents, their set is defined as $S_{A}\in \left\{1,2,\ldots ,N\right\}$, each of the agents is denoted by *i*, and each has a state $x_{i}\in R^{2}$ in a two-dimensional area *Q*. The collective states of the multi-agent system are denoted as the set of $x=\left\{x_{1},x_{2},\ldots ,x_{N}\right\}\in R^{2\times N}$. Therefore, the coverage task is defined as placing the agents in the given area so that the agents can occupy the entire area equally and uniformly. For the coverage task, we can establish the objective function from Cortes [37] as follows:

(1) $$J_{\mathrm{cov}}\left(x\right)=\int_{Q}\;\min_{i\in \{1,2,3\ldots ,\ell (x)\}}f_{\mathrm{uc}}(∥q-x_{i}∥)f_{\mathrm{wt}}\left(q\right)dq,$$

where ℓ(x) denotes the length of *x* that is equal to the number of agents *N* considered, a bounded set $Q\subset R^{2}$ describes the respective environment of agents, function $f_{\mathrm{uc}}:R_{0+}\to R_{0+}$ associates the utility cost of positioning an agent at a distance from a given location *q* in *Q*, and the relative weight $f_{\mathrm{wt}}:R^{n}\to R_{0+}$ denotes the quantified significance of any point q∈Q. With a reasonable sized *Q*, the coverage can be considered to be achieved at $x^{*}$ when $J_{\mathrm{cov}}\left(x\right)$ has a minimum value, i.e., the coverage control problem is to place the agents that minimize $J_{\mathrm{cov}}\left(x\right)$.

The equation of the state in the discrete time framework at time *t* of any individual agent i=1,2,3,…,N can be determined as follows:

(2) $$x_{i}\left(t+1\right)=x_{i}\left(t\right)+u_{i}\left(t\right),$$

where at *t*, the agent’s position is $x_{i}\left(t\right)\in R^{2}$, and the control input is $u_{i}\left(t\right)\in R^{2}$. According to [35], each agent communicates its current and intended states to the GC, and based on the collective agents’ state x(t), respective scalar feedback to all the agents is provided by the (single) global coordinator (GC).

Particularly, at every time step, each agent intends to take an action as follows:

(3) $${\hat{u}}_{i}\left(t\right)=\gamma_{c}\left(t\right)\delta_{i}\left(t\right),$$

where $\gamma_{c}$ is a gain parameter, and $\delta_{i}\in {\left\{-1,1\right\}}^{2}$ is a random parameter, which is generated with equal probability based on the Bernoulli distribution having a ±1 value. Thus, with the intended action of the agent ${\hat{u}}_{i}\left(t\right)$, the predicted state of agent *i* becomes

(4) $${\hat{x}}_{i}\left(t\right)=x_{i}\left(t\right)+{\hat{u}}_{i}\left(t\right).$$

The performance objectives $J_{\mathrm{cov}}\left(x\right)$ and $J_{\mathrm{cov}}\left(\hat{x}\right)$ for $\hat{x}=\left\{{\hat{x}}_{1},{\hat{x}}_{2},\ldots ,{\hat{x}}_{N}\right\}$ at *t* can be determined by the current and intended states ($x_{i}\left(t\right),{\hat{x}}_{i}\left(t\right)$) of each agents when they communicate with the GC; thus, the broadcast feedback signal F(t) becomes

(5) $$F\left(t\right)=J_{\mathrm{cov}}\left(\hat{x}\left(t\right)\right)-J_{\mathrm{cov}}\left(x\left(t\right)\right)$$

In a 2D space, $J_{\mathrm{cov}}:R^{2N}\to R_{0+}$ evaluates a motion coordination task. The local controller of each agent *i* executes the control action once they receive the feedback signal from the GC as follows:

(6) $$u_{i}\left(t\right):=-\alpha \left(t\right)\frac{F(t)}{\gamma_{c}\left(t\right)}{{\hat{u}}_{i}}^{\left[-1\right]}\left(t\right),$$

where ${{\hat{u}}_{i}}^{\left[-1\right]}$ denotes the element-wise inverse, and α(t) denotes the gain parameter (equivalent to a learning factor). The gain parameters α and $\gamma_{c}$ must satisfy certain criteria [26] to ensure convergence, such as

(7) $$\left\{\begin{array}{c} \lim_{t\to \infty }\alpha \left(t\right)=0,\;\\ \sum_{t=0}^{\infty }\alpha \left(t\right)=\infty ,\;\\ \lim_{t\to \infty }\gamma_{c}\left(t\right)=0,\;\\ \sum_{t=0}^{\infty }{\left(\alpha \left(t\right)/\gamma_{c}\left(t\right)\right)}^{2}<\infty ,\\ \sum_{t=0}^{\infty }\alpha \left(t\right)\gamma_{c}^{2}\left(t\right)<\infty .\; \end{array}\right.$$

By ensuring the above characteristics of the user-defined gain parameters, α and $\gamma_{c}$ can be designed typically as [26]

(8) $$\alpha \left(t\right)=\frac{a_{0}}{{\left(t+1+a_{1}\right)}^{a_{2}}}$$

(9) $$\gamma_{c}\left(t\right)=\frac{c_{0}}{{\left(t+1\right)}^{c_{1}}},$$

where the parameters $a_{0},a_{1},a_{2},c_{0},c_{1}$∈$R_{+}$ are chosen arbitrarily to satisfy (7).

The decision (14) is unrestricted, and depending on the gradient and gain parameters, the magnitude of $u_{i}\left(t\right)$ can be unrealistically very large. Therefore, with a realistic limit given by $d_{\mathrm{mx}}$, the actual decision can be modified as

(10) $$u_{i}\left(t\right):=d_{\mathrm{mx}}\tanh\left(-\frac{\alpha (t)}{d_{\mathrm{mx}}}\frac{F(t)}{\gamma_{c}\left(t\right)}{{\hat{u}}_{i}}^{\left[-1\right]}\left(t\right)\right),$$

According to Azuma and Ito [26,35], assuming a sufficiently large $d_{\mathrm{mx}}$, the convergence criteria given for the stochastic optimization of a multi-agent system is guaranteed while satisfying through the reparation of actions with the gain factors (8) and (9), thus yielding

(11) $$\lim_{t\to \infty }J_{\mathrm{cov}}\left(x\left(t\right)\right)=\min_{x\in R^{2N}}J_{\mathrm{cov}}\left(x\right).$$

As time approaches infinity, the agent’s states reach a point where the objective function value is minimum, i.e., the multi-agent system reaches an optimal point. For the details on the convergence proof, they can be referred to in [26,35].

### 2.2. Multi-Coordinator-Based Distributed BC Scheme

Figure 1 juxtaposes the typical BC and the concept of the proposed DBC with hierarchical coordination. For the multi-coordinator-based DBC scheme elucidated in Figure 1b, it is comprised of hierarchical (bi-level) broadcast signals: the higher level (from global coordinator to sub-coordinator agents) and lower level (from sub-coordinators to the respective ordinary agents). In the DBC scheme, among *N* agents, few agents are preset to be sub-coordinators to play dual roles: they coordinate other agents and serve coverage as ordinary agents. Assume that a subset of agents $S_{C}\subset \left\{1,2,\ldots ,N\right\}$ constitutes sub-coordinators that serve dual roles. The BC takes place at the higher and lower levels ascribed earlier. Note that any agent can be selected as an SC, and for the proper coverage by SCs given by the Voronoi diagram, at least three SCs are required. In real cases like robots or UAVs, only agents equipped with the necessary communication and computation tools to perform a dual role can be selected as SCs.

Specifically under this distributed technique, the sub-coordinator agents and GC-based BC operate at the higher level, as previously described. In particular, in this method, the performance index, the state update, and the state prediction defined by (1)–(4), respectively, remain unchanged. However, the GC’s coverage evaluation for creating the broadcast signal differs depending on the number of SCs, that is, coverage by the SC agents is assessed using their aggregate states $y=\{x_{m},\;\forall m\in S_{C}\}$. Specifically, the collective states of the SC agents, and based on their current y(t) and predicted state $\hat{y}\left(t\right)$, the GC determines the broadcast signal as

(12) $$F_{\mathrm{GC}}\left(t\right)=J_{\mathrm{cov}}\left(\hat{y}\left(t\right)\right)-J_{\mathrm{cov}}\left(y\left(t\right)\right).$$

Note that although the coverage function $J_{\mathrm{cov}}\left(\cdot \right)$ is the same, $J_{\mathrm{cov}}\left(x\right)$ and $J_{\mathrm{cov}}\left(y\right)$ are different due to the different number of agents reflected by the size of states *x* and *y*, respectively.

Each SC agent *m* is considered to have an operational range of radius $R_{\mathrm{sc}}$, and all agents within it are coordinated via local broadcast signals. Assume that the agents’ state under SC *m* is provided by $z^{m}=\{x_{k},\;\forall k\;s.t.\;|x_{m}-x_{k}|\leq R_{\mathrm{sc}}\}\subset x$, and the corresponding local set of agents is given by $S_{A}^{m}=\left\{\forall k\;s.t.\;\right|x_{m}-x_{k}|\leq R_{\mathrm{sc}}\}$.

Each SC *m* collects the states of the local agents $z^{m},{\hat{z}}^{m}$ and offers a scalar feedback $F_{\mathrm{sc}}^{m}$ to them as

(13) $$\begin{array}{ccc} F_{\mathrm{sc}}^{m}\left(t\right) & = & J_{\mathrm{cov}}\left({\hat{z}}^{m}\left(t\right)\right)-J_{\mathrm{cov}}\left(z^{m}\left(t\right)\right),\\ & \; & \;\forall q\in Q\;s.t.\;|q-z_{m}|\leq R_{\mathrm{sc}}. \end{array}$$

This means that an SC only considers the region represented by $q\in Q\;\forall |q-z_{m}|\leq R_{\mathrm{sc}}$ for the partial coverage evaluation.

Finally, the local controller obtains and performs all agents’ deterministic actions, including SC agents. Specifically, each agent *i* with the role of SC (i.e., $i\to m\in S_{C}$) determines the action using both feedback signals from the GC and the SC *m* itself as

(14) $$v_{i}\left(t\right):=-\alpha \left(t\right)\frac{\omega F_{\mathrm{GC}}\left(t\right)+\left(1-\omega \right)F_{\mathrm{sc}}^{m}\left(t\right)}{\gamma_{c}\left(t\right)}{{\hat{u}}_{i}}^{\left[-1\right]}\left(t\right),\;\forall i\in S_{C}$$

where $\omega_{i}\in \left(0,1\right)$ is the weight corresponding to the trade-off between the upper and lower coverage objectives. As ordinary agents ($\forall i,i\notin S_{C}$) may receive feedback from multiple SCs, they determine their actions as

(15) $$v_{i}\left(t\right):=\frac{-\alpha (t)}{\sum_{k=1}^{K}w_{k}}\frac{\mu \sum_{k=1}^{K}w_{k}F_{\mathrm{sc}}^{k}\left(t\right)}{\gamma_{c}\left(t\right)}{{\hat{u}}_{i}}^{\left[-1\right]}\left(t\right),\;\forall i\notin S_{C}$$

where *k* denotes the SC, μ is the tuning factor, and $w_{k}$ is a weighting factor. Note that the agent determines its action based on the weighted average from *K* feedback signals, and the weight $w_{k}$ is tuned according to the distance from each SC *k*. Note that with μ=1 and a sufficiently large $R_{\mathrm{sc}}$ to cover the entire area and all agents by any SC, the decision (15) becomes exactly the same as (14) (as the average feedback will be equal to the single coordinator’s feedback). In this study, we evaluated the proposed DBC with (0<β<1) and without (β=0) the momentum term by referring to them DBCm and DBC for simplicity, as described in the next section.

Since the feedback with a partial view by the SC can be noisy the control input is finally determined by introducing a *momentum* factor β∈(0,1) with the previous estimation of action $v_{i}\left(t-1\right)$ for each of the agents as

(16) $$u_{i}\left(t\right)=d_{\mathrm{mx}}\tanh\left(\frac{1}{d_{\mathrm{mx}}}\left(\beta v_{i}\left(t-1\right)+\left(1-\beta \right)v_{i}\left(t\right)\right)\right),$$

where $d_{\mathrm{mx}}$ represents a limiting factor with the hyperbolic tangent function that restricts the maximum moving step each time. The gain parameters $\alpha ,\gamma_{c}$ are tweaked similarly to the original BC described above (8) and (9), and through a similar repeated process, the agents find a state that minimizes the overall objective function.

## 3. Numerical Simulation and Evaluation

The proposed DBC scheme availing multiple and bi-level coordinators was appraised through numerical simulation for a coverage task in an enclosed environment of 80×120, with an initial placement of 60 agents posited closely in the lower left corner and using only 10% of the total area, i.e., they need to move effectively to also spread over the rest of the 90% area. It is a more difficult task than [26], where only seven agents were initially spread over 60% of the total area. Furthermore, each agent was restricted to a maximum travel distance of $d_{\mathrm{mx}}=3$ units per iteration. The gain parameters corresponding to α and $\gamma_{c}c$ were set for optimal performance using the existing BC scheme. Specifically, both gain parameters were set to $a_{0}=2,a_{1}=15,a_{2}=0.73,c_{0}=1,c_{1}=0.65$. In addition, the momentum factor was set as β=0.5, and the trade-off parameter was set as ω=0.9 for the SC, thus implying a higher weight being applied to the global feedback. For a fair comparison, the tuning parameter μ=0.9 was considered to approximately realize the same average travel distance by the agents compared with the BC. Furthermore, the range of the distributed sub-coordinators was defined by a circular region with a radius of $R_{\max}=40$ units, which enabled them to obtain information about any other agents within their line of sight. Hence, for the given $R_{\max}=40$, the weight $w_{k}$ was tuned as a function of distance (from the SC to an agent), with $w_{k}=1$ for a distance from 0 to 35, which ($w_{k}$) linearly decreased from 1 to 0 from a distance of 35 to 40.

**Figure 2:**
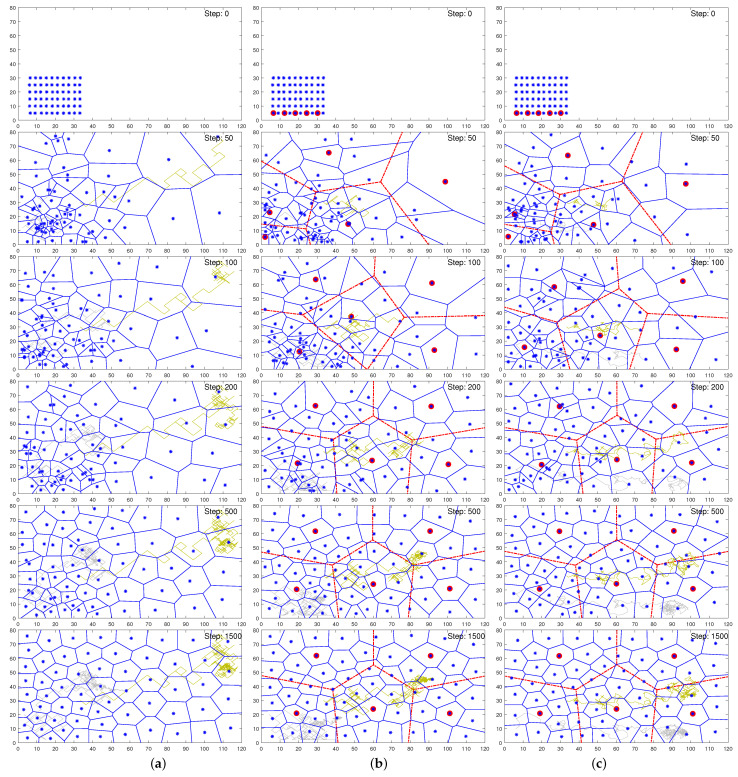
Coverage performance (Voronoi with blue lines) comparison of (**a**) BC, (**b**) DBC, and (**c**) DBCm at steps 0, 100, 200, 500, and 1500 from top to bottom, respectively. For DBC and DBCm, agents with red circles are the sub-coordinators, and the red Voronoi (with red dasehed lines) shows their higher level coverage using only the SCs. The light gray and yellow lines in the figures show the footprints of two arbitrary agents.

With the above set parameters, the multi-agent system was numerically simulated for the coverage task employing the typical single-coordinator-based BC scheme and the proposed DBC and DBCm schemes. Figure 2a–c depict the cases of the typical BC and the proposed DBC and DBCm schemes, respectively, by showing the respective agents’ positions in the environment using the Voronoi diagrams corresponding to iteration steps 0, 50, 100, 200, 500, and 1500 ordering from top to bottom.

The top schematic in Figure 2 shows the initial states of the agents. In Figure 2b,c, five sub-coordinators are shown with red marks, and they performed dual roles: (1) serving the role of an ordinary agent to achieve coverage goals and (2) coordinating their neighboring agents. It can be seen that within iteration step 100, the SCs were well distributed, as shown by the red Voronoi diagram in the 2nd to 6th ones of Figure 2b,c, thus resulting in superior distributions of the other agents as well. Not much significant difference in agent coverage was observed at this stage, as in each case, the constraint of maximum moving distance remained active. Subsequently, at 200 steps, the agents in DBC or DBCm covered the area better than in BC. At this stage, BC showed a much larger coverage cells of agents on the right side than on the left side due to inadequate coordination and movement restriction per step. After 500 steps, some agents still remained very close in BC, as found on the left side, whereas coverage was almost apparent for the proposed schemes. However, the discrepancy reduced with BC at higher steps. Later, at 1500 steps, the difference was found to be marginal compared to the earlier steps. Overall, the typical single-coordinator-based BC scheme was incongruously scattered at the end of 1500 steps and proceeded slowly. Therefore, these results illustrate that distributed coordination can make coverage faster for the sub-optimal state.

In addition, these graphs also include the path trajectories or footprints of two arbitrary agents (as examples) to demonstrate the better visibility of the dispersion throughout the iterations. Specifically, the light gray and yellow lines in the figures depict the travel paths of the second agent from the bottom left corner and the second last agent from the top right corner from the initial placement. The gray and yellow lines from Figure 2a show that both of the agents leaped with large steps at the beginning to dispatch faster and ended up oscillating at their final positions. It is apparent that DBC also showed a similar pattern for the same agents. However, agents in DBCm are often headed toward the momentum of previous steps and gradually reduce their step size with a better and smoother path to the final point. Despite their same initial position, the agents eventually move to different points for these methods due to the highly stochastic nature of the decision algorithm. Nevertheless, DBCm trumped both the BC and DBC schemes shown in Figure 2c, thus depicting remarkably uniform steps apropos to smoother convergence. Therefore, it is expected that DBCm can reduce the energy, execution time, or costs associated with the distance traveled by the agents while converging.

**Figure 3:**
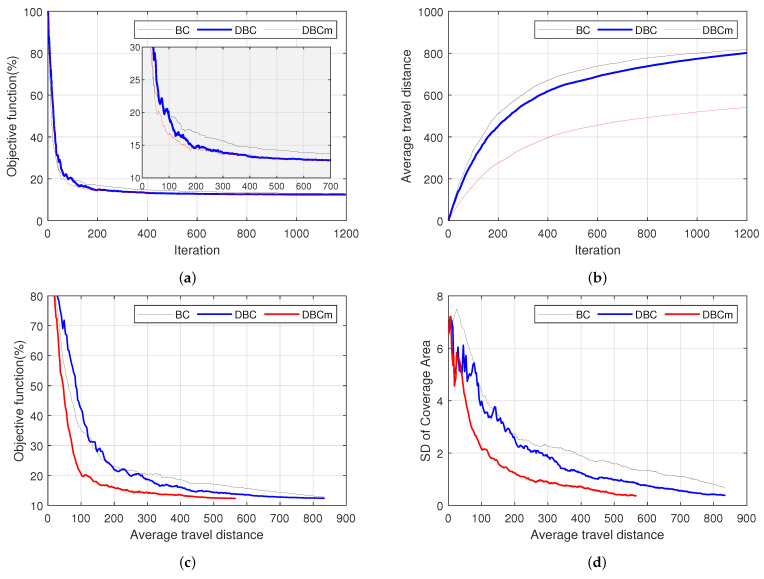
Performance comparison of three schemes (BC, DBC, and DBCm): (**a**) objective function, (**b**) average travel distance per agent, (**c**) average travel distance (travel cost) vs. objective function, and (**d**) average travel distance vs. standard deviation of coverage areas.

Figure 2 only visualizes the coverage task and the process without providing a quantitative indication of the degree of task completion. As the performance index gives the quantitative measure of the task objective, its low value during the coverage progress with no further (or negligible) changes indicates task completion. Therefore, the coverage visualized in Figure 2 was further analyzed quantitatively in terms of their convergence to the final states using the performance index. Note that there can be large possible combinations of agents for optimal coverage, which is unknown in this case, and therefore, the mean square error from the target points cannot be obtained to understand the convergence or task accomplishment. As is usual practice in the literature [31,33,35,41], we have examined the convergence in terms of the value of the objective function. Particularly, Figure 3a shows the convergence graphs of three schemes in terms of the percentage of the objective values concerning their maximum value (obtained at the initial states of the agents). To enhance visualization, a close view of the plots is shown for 0 to 700 steps, thus demonstrating that DBC and DBCm reached the near-optimal point much earlier than the traditional BC model, albeit their final convergence values appear almost the same.

More importantly, Figure 3b shows the cumulative average trip distance per agent with respect to the number of iterations. BC agents required the longest distances, followed by DBC, which was imperceptibly better at early iterations but ended up with the same value. It is worth noting that the growing trends in the distance traveled by DBC can be amended by halting them after convergence or properly tweaking the gain function’s parameters. Consequently, DBCm showed a minimum moving distance throughout the iteration period. In contrast to previous studies [26,35], we have observed two additional evaluation criteria that are shown in Figure 3c,d. For any real implementation of a multi-agent system, it is necessary to minimize the operating energy, which is directly related to the travel distances. Here, Figure 3c delineates the travel costs in terms of the travel distance by an agent and the task accomplishment in terms of the objective function. It is clear from these results that the proposed DBC schemes are both cost-effective and time-saving in achieving the goal of the multi-agent coverage task. For instance, there were 60 agents in this coverage task (in 100% area), and the average coverage per agent was fixed at about 1.67%. Since the coverage areas of each agent were not identical, we observed the standard deviation of the coverage areas of the agents with respect to the travel distance shown in Figure 3d. Based on the figures, the proposed methods unprecedentedly outperformed in reducing the standard deviation and minimizing the traveling distance.

**Figure 4:**
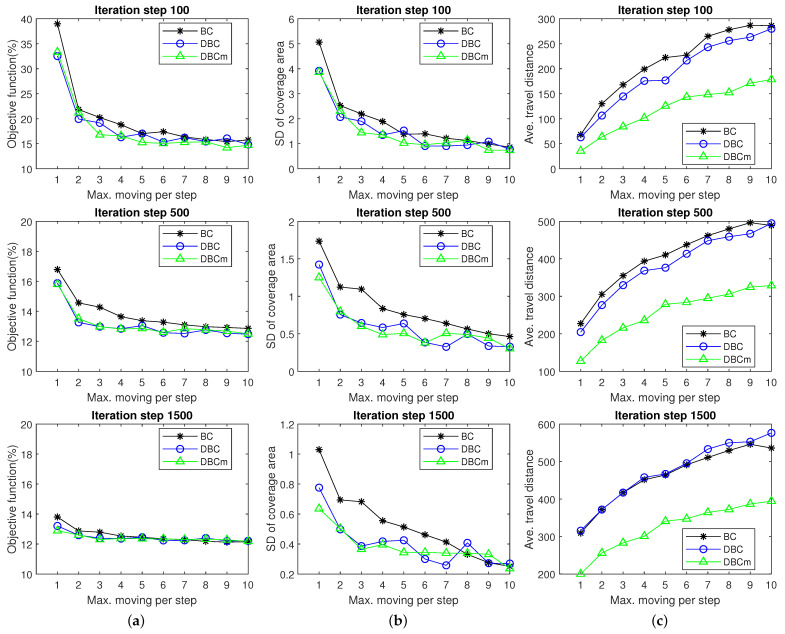
Performance comparison of the schemes (BC, DBC, and DBCm) for the constraint parameter $d_{mx}$ related to the maximum moving per step verses (**a**) objective function, (**b**) standard deviation of the coverage area, and (**c**) average travel distance of the agents, all observed at iterations 100, 500, and 1500.

Originally, the BC schemes were designed with unrestricted moving distance per step. As a result, with large moving steps, agents disperse over the operating area and then gradually prone to settle toward the coverage points quickly. If the agents are restricted to a limited maximum distance per step, their performance also notably changes. The size of the distance provides different outcomes for the iteration and the distance they travel to achieve the target. Once they are restricted to a limited maximum distance per step, their performance notably varies. Here, a detailed performance evaluation was conducted for different moving step sizes related to parameter $d_{\mathrm{mx}}$ from 1 to 10, and the convergence performance outcomes at 100, 500, and 1500 can be observed for these methods, as shown in Figure 4. Particularly, Figure 4a shows that at the 100th iteration, the objective function values greatly diverged with the maximum moving step. Particularly, with larger steps, these methods reduced the objective function conspicuously as they converged faster. However, at the 1500th iteration, their differences became nominal. Remarkably, DBC and DBCm exhibited better performance outcomes than BC at the early iterations when the value of $d_{\mathrm{mx}}$ was smaller. After examining Figure 4b, the standard deviation of the convergence area was found to be greatly reduced by DBC and DBCm with smaller values of $d_{\mathrm{mx}}$. At the same time, a similar pattern is observed for the case of traveling distance in Figure 4c. In the concluding remark, the proposed method has been found to be better with faster convergence towards a sub-optimal point even when the agents are restricted to a maximum movement per step. In the real world, there are many multi-agent applications where only a sub-optimal decision is sufficient within a very short time, where this method can be effectively applied. An example of such a system is traffic control in road networks using BC [41], where a quick sub-optimal gap between the vehicles is necessary instead of a time-consuming decision to have precise optimal gaps. The proposed hierarchical coordination-based DBC is expected to be effective in controlling a larger traffic network than the case in [41] in the future connected/automated transportation. We aim to extend the present work for such a traffic coordination task and evaluate DBC in the future.

## 4. Discussion

**Figure 5:**
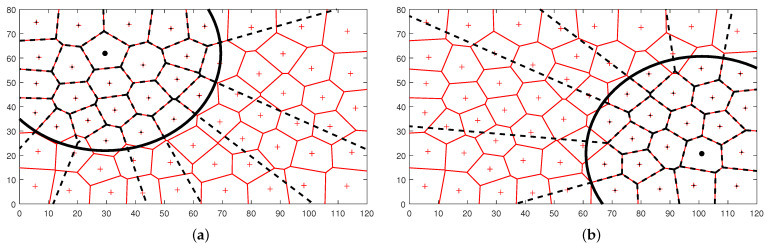
The global coverage (red Voronoi diagram) and local coverage under SCs (black dashed Voronoi within the black circle). The operative areas under two distinctly located SCs are shown using black circles in (**a**,**b**) for the same global coverage of the agents.

As a pioneer approach, DBC has been proposed in this paper, and several aspects need to be discussed further. The above results are shown in this study for the case of five SCs, which were quickly dispersed over the area under the coordination of GC. The coverage objectives under the SCs, with varying numbers of agents and changing areas, differed from the global coverage objectives (for all agents and entire areas), as given in Figure 5. Figure 5a,b show regions under two different SCs using black circles. Most inner agents had the same Voronoi cells as the global ones. Therefore, the change in the local coverage objective values due to an agent movement under an SC is expected to have the same trends as the global coverage objectives. This is why local actions by agents in DBC eventually provide a global coverage solution. However, some agents near the boundary (circle) had slightly different shapes and may have noisy or conflicting information from one SC, which is highly likely to be compensated by the information from the other SCs, since the agents near the boundary may also fall under nearby SCs.

The selection of the SCs and operating range can also be justified from the above, which is also related to the operating stability of the proposed DBC. Specifically, the SCs should be selected to collectively cover the total environment, thus allowing for some overlapping parts to facilitate the agents near the respective boundary to properly shift from under one SC to another for complete and uniform coverage. In principle, the systems considered are stable, i.e., without any external control, they do not show unstable behavior even in non-equilibrium states due to noise. Therefore, the stability entirely depends on the applied control inputs to the agents, i.e., whether the system converges/diverges, remains stable, or becomes unstable depends on the way that the control input is selected. The criteria (e.g., use of small gain parameters with decreasing values) of such convergence under the stable operation of general stochastic optimization are described in (7)–(11). Note that each optimization under DBC has the same gain parameter settings required for the convergence. The only difference is whether the weighted gradient (15) in DBC is reliable or provides smooth gradients (without discontinuity) for a smooth operation of the agents, which depends on the number of SCs and their coverage. To ensure that the weighted gradient is close to the true gradient (in terms of their magnitude approximately) in the global view, we provides sufficient overlapping of the SCs’ ranges and tuned the additional gain parameters of DBC by closely observing and comparing the optimization process. Although the smaller region per SC can keep the computational complexity of the distributed SCs low, a trade-off is necessary to ensure stability and convergence in DBC, which we dealt with conservatively in this first-ever study.

This paper used coverage control, a well-studied and easy-to-understand benchmark problem for the class of multi-agent systems, to demonstrate the underlying concepts and effectiveness of DBC. However, DBC (like BC) is merely a model-free stochastic optimization mechanism that can be applied to any system optimization that retains the agents’ autonomous behavior, i.e., agents can flexibly choose an action without restrictions. The road traffic system is one of the ideal examples of such a multi-agent system, where each vehicle takes independent action, signaling systems choose the red–green time independently, and their collective behavior determines the quality of the transportation system. Using a few agents, BC was applied to coordinate the merging of vehicles on the highway, thus applying them only when they were close to the merging point, since the optimization needed to be converged quickly [41]. Now, DBC can be used for a more extended range with more merging vehicles coordinating effectively in the same scenarios. BC was also applied independently in a receding horizon control scheme to determine the optimal duration of traffic lights at an intersection without coordinating with the other intersections [42]. The proposed DBC can be applied to coordinate multiple intersections simultaneously, where each intersection can be coordinated by an SC, and a GC can coordinate all the SCs. Such exciting applications in the future of connected automated traffic paradigms will be worth investigating, and DBC tailored to such systems will be investigated in our future work.

## 5. Conclusions

The BC scheme is one of the novel strategies unlocked in the swarm robot control field, which has been extended to drive a larger multi-agent system more efficiently in obtaining the global goal. Specifically, this paper has articulated the fundamental principle and established the mathematical model of employing multiple coordinators with hierarchy in the BC scheme. According to the scheme, some agents perform dual roles in making the multi-agent system more efficient by ensuring better-coordinated behavior. The proposed DBC scheme showed much better performance, as it could reach the near-optimal states quickly with better coordination using distributed feedback. The outcomes, in terms of the convergence speed, the travel cost of agents, and the cost-effectiveness, show that the proposed DBC scheme is realizable to have better performance than the existing BC schemes in solving various real-world multi-agent systems. The proposed BC method is suitable for the case where the near-optimal value of the objective function within the shortest time is sufficient. As we are getting very close to the target in the shortest possible time and with the smaller average traveling distance of the agents, the energy cost is less as well. For the agents that are used in emergency operations, a faster response is very much crucial, and this method will be effectively applicable there.

The proposed DBC scheme is anticipated to be another step toward integrating cutting-edge technology in managing large-scale autonomous systems, such as connected autonomous vehicles in traffic networks, which would be our exciting and challenging future work. 

## Data Availability

The original contributions presented in the study are included in the article, further inquiries can be directed to the corresponding authors.
